# Collagen-Based Electrospun Materials for Tissue Engineering: A Systematic Review

**DOI:** 10.3390/bioengineering8030039

**Published:** 2021-03-18

**Authors:** Britani N. Blackstone, Summer C. Gallentine, Heather M. Powell

**Affiliations:** 1 Department of Materials Science and Engineering, The Ohio State University, Columbus, OH 43210, USA; Blackstone.38@osu.edu; 2 Department of Biomedical Engineering, The Ohio State University, Columbus, OH 43210, USA; gallentine.5@osu.edu; 3 Research Department, Shriners Hospitals for Children, Cincinnati, OH 45229, USA

**Keywords:** collagen, electrospinning, tissue engineering

## Abstract

Collagen is a key component of the extracellular matrix (ECM) in organs and tissues throughout the body and is used for many tissue engineering applications. Electrospinning of collagen can produce scaffolds in a wide variety of shapes, fiber diameters and porosities to match that of the native ECM. This systematic review aims to pool data from available manuscripts on electrospun collagen and tissue engineering to provide insight into the connection between source material, solvent, crosslinking method and functional outcomes. D-banding was most often observed in electrospun collagen formed using collagen type I isolated from calfskin, often isolated within the laboratory, with short solution solubilization times. All physical and chemical methods of crosslinking utilized imparted resistance to degradation and increased strength. Cytotoxicity was observed at high concentrations of crosslinking agents and when abbreviated rinsing protocols were utilized. Collagen and collagen-based scaffolds were capable of forming engineered tissues in vitro and in vivo with high similarity to the native structures.

## 1. Introduction

Collagen, specifically type I, is a major constituent of many tissues and organs, including skin, bone, tendon, blood vessels, and cardiac tissue. As a result, collagen type I matrices are often used as a surrogate extracellular matrix (ECM) for in vitro tissue engineering and in vivo tissue regeneration or repair [1,2,3,4,5]. Given the fibrous nature of the native ECM, electrospinning, a technique that creates matrices comprised of nanometric or micron-sized fibers, is commonly utilized to generate scaffolds for tissue engineering [6,7,8,9]. Scaffold properties, such as fiber diameter, porosity, interfiber distance, and fiber organization can be altered via changes in spinning parameters (polymer, solvent, solution concentration, needle-ground distance, applied voltage), leading to a wide variety of scaffold architectures that can be manufactured to mimic the native ECM structure [10,11]. Additionally, mechanical properties and resistance to degradation can be tuned via crosslinking and/or through the incorporation of higher strength materials to the spinning process [12,13,14,15,16].

With the vast literature available on electrospun collagen materials, it can be challenging to determine the most critical fabrication parameters, the connection between these parameters and functional outcomes, and subsequently, the level of biomimicry required for successful tissue regeneration. In addition, there are conflicting reports regarding the ultrastructure of electrospun collagen materials [17,18], the toxicity of chemical crosslinkers used to stabilize the matrices [19,20] and mechanical properties of these matrices [21,22]. Thus, the objective of this systematic review was to examine the literature on electrospun collagen materials for tissue engineering to examine trends in source materials, electrospinning parameters and crosslinking techniques and to determine if any connections exist between these parameters and functional outcomes.

## 2. Materials and Methods

**Figure 1:**
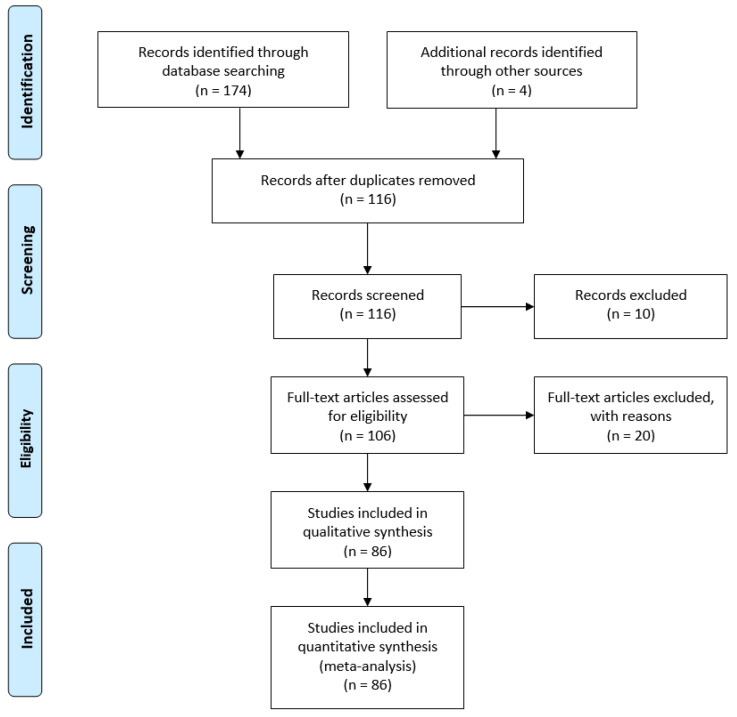
Flow-through diagram for literature search and inclusion/exclusion criteria.

A systematic review of the literature published on electrospun collagen materials for tissue engineering applications was conducted. PubMed and Web of Science were searched for “electrospun collagen” AND “tissue engineering” with duplicates removed. One hundred thirteen articles were assessed for inclusion in the analysis. Articles were excluded if not available in English. Reviews, book sections, conference proceedings and patents were also excluded. Inclusion was determined by relevance to the topic (i.e., must examine collagen-containing electrospun materials for tissue engineering/regenerative medicine applications) (Figure 1). After assessment [16,17,19,20,21,22,23,24,25,26,27,28,29,30,31,32,33,34,35,36,37,38,39,40,41,42,43,44,45,46,47,48,49,50,51,52,53,54,55,56,57,58,59,60,61,62,63,64,65,66,67,68,69,70,71,72,73,74,75,76,77,78,79,80,81,82,83,84,85,86,87,88,89,90,91,92,93,94,95,96,97,98,99,100,101,102], studies met the selection criteria and were considered in this review (Supplemental Table S1). The objective of this systematic review was to assess the role of collagen source, electrospinning parameters (solvent, solution composition) and crosslinking method on the resultant materials properties and their utility for tissue engineering. Outcomes for each paper were assessed independently by all authors (BNB, SCG and HMP). For quantitative analyses on material composition, source, and crosslinking method, all 86 papers were included. If multiple collagen sources, compositions and/or crosslinking methods were used in a single manuscript, each unique feature was included individually in the total count. For example, if one manuscript examined scaffolds electrospun using a blend of collagen type I and polycaprolactone (PCL) at multiple ratios, including a collagen only group, it was counted as a citation for pure collagen and as a citation for PCL-collagen blends (different ratios of the same polymers were counted only once).

## 3. Results

### 3.1. Sources of Collagen for Electrospinning

**Figure 2:**
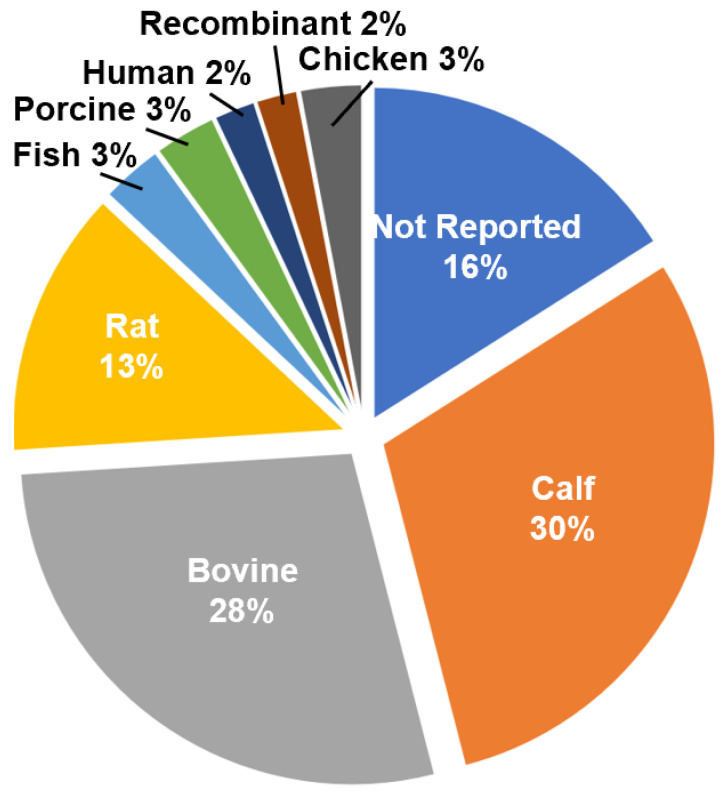
Distribution of collagen sources utilized in the study cohort for electrospinning.

Collagen can be extracted from a number of different tissues and a wide variety of organisms, including mammals, amphibians, fish, and birds [103]. For use with electrospinning, collagen from bovine and calf dermis dominates the literature, followed by collagen of rat origin (Figure 2). The frequent use of bovine and calf collagen is likely due to the abundance of the source tissue and commercial availability of the isolated collagen. Due to concerns regarding potential allergen or pathogen risk from animal sources, many recombinant technologies to produce collagen, specifically type I, have been developed. Genetic modification of tobacco plants with two genes encoding recombinant heterotrimeric collagen type I and the human prolyl-4-hydroxylase (P4H) and lysyl hydroxylase 3 (LH3) enzymes has been shown to result in the formation of plant-extracted rhCOL1, which forms thermally stable triple helical structures [104]. Though these recombinant materials are commercially available, they are not widely used, comprising only 1% of the total available manuscripts on electrospun collagen (Figure 2).

### 3.2. Ultrastructure of Electrospun Collagen

**Figure 3:**
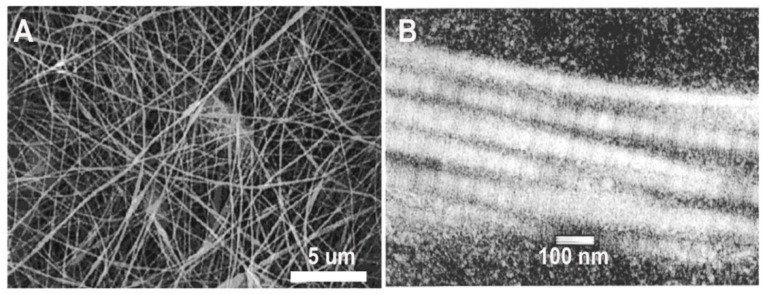
(**A**) SEM image of a scaffold electrospun from calfskin collagen type I solubilized in 1,1,1,3,3,3-hexafluoro-2-propanol (HFP). (**B**) TEM image of an individual electrospun collagen fiber exhibiting D-banding. Modified from [22] with permission.

In order to utilize electrospinning to fabricate collagen scaffolds, generally, some level of denaturation of the collagen is required to solubilize the collagen and yield a solution capable of being electrospun [18]. The solubilization process is often thought to denature the protein to the extent that the D-banding observed with native collagen type I is lost. This banding is seen at the quaternary structure level, where collagen type I fibrils are formed by monomers assembled end to end and aligned in a parallel and staggered fashion, creating light and dark bands observed using transmission electron microscopy. This structure is critical to the mechanical properties of native collagen [105,106], though it is often believed to be absent in electrospun collagen fibers [36]. Lack of D-banding is commonly associated with the type of solvents used for electrospinning, specifically the widely used fluoroalcohol family of solvents, such as 1,1,1,3,3,3-hexafluoro-2-propanol (HFP) and 2,2,2-trifluoroethanol (TFE) [18]. Using a combination of circular dichroism (CD) and Fourier-transform infrared spectroscopy (FTIR), Bürck et al. found that HFP and TFE nearly completely unfolded the triple helixes of collagen type I derived from bovine dermis [107]. Though electrospinning restored up to 38% of the polyproline fraction, no D-banding was detectable in these fibers using TEM [107]. The absence of D-banding was also observed in electrospun collagen type I from bovine dermis [36], the porcine dermis [64], and tilapia skin [59], all solubilized in HFP. Within the literature, almost 70% of studies utilized HFP as a solvent for collagen when electrospinning. However, most of these studies did not examine the ultrastructure of these materials, with only eleven manuscripts [17,22,25,27,30,36,38,59,64,86,93] out of the 86 examined reporting on D-banding. Of these studies, six reported evidence of D-banding in their materials [17,22,25,27,38,86]. While overall, few studies have reported D-banding with HFP, Matthews et al. found D-banding in fibers electrospun from solutions of acid-soluble, lyophilized type I collagen from calfskin (83 mg/mL; Sigma-Aldrich, St. Louis, MO, USA), dissolved in HFP and delivered at a flow rate of 5 mL/h (Figure 3A,B) [22]. Collagen D-banding was also observed via atomic force microscopy in collagen-PCL blends solubilized in HFP [25]. Though the use of HFP as a solvent is conventionally thought to substantially denature collagen with little to no recovery of the structure during fabrication, the use of HFP with type I collagen isolated from calfskin results in fibers with D-banding [17,22,25,27,86]. Thus, the raw collagen material, including the source and isolation process, is an important in maintenance of ultrastructure (Table 1).

Other solvents have been investigated as potential solubilizers of collagen with the goal that less of the original structure is lost during solubilization. Fish-derived collagen type I (Helisorb^®^, Medira Ltd., Cambridge, England) was solubilized in a 93/7 ratio of glacial acetic acid/dimethyl sulfoxide at 10% weight/volume [38]. A flow rate of 0.60 mL/h resulted in fibers ranging in diameter from 200 nm to 1100 nm and displaying D-banding [38]. Improvements in structure resulting from changes to aqueous solvent, however, come with some drawbacks. The slower evaporation rate of acidic solutions, when compared to fluoroalcohols, requires much slower flow rates, limiting the fiber size and collection over time. Additionally, fibers are more likely to be wet when deposited and join with other fibers around them, decreasing porosity and potentially altering mechanical behavior.

### 3.3. Methods to Enhance the Stability and Mechanical Properties of Electrospun Collagen: Crosslinking and Composition

#### 3.3.1. Crosslinking

While electrospun collagen is a desirable material for tissue engineering scaffolds because of its architectural versatility and biocompatibility, the changes in the structure that occur during processing can result in scaffolds with high degradation rates and mechanical properties that are insufficient for many applications. Electrospun collagen scaffolds rapidly degrade in aqueous environments [36] and can be orders of magnitude weaker than native collagen, limiting in vitro culture, handleability during implantation, and resistance to in vivo forces. Chemical and physical crosslinking electrospun collagen has been shown to improve both scaffold stability and mechanical properties (Table 2).

Chemical crosslinking methods with agents, such as glutaraldehyde, carbodiimide, genipin, 1,4-butanediol diglycidyl ether (BDDGE) and transglutaminase are widely used and have significant effects on scaffold properties. Glutaraldehyde is among the most widely used chemical crosslinker for collagen-containing electrospun materials and rapidly facilitates covalent bonding between lysine residues in the collagen molecules. Immersion in glutaraldehyde solutions as dilute as 0.25% was sufficient to provide stability for four weeks of in vitro culture followed by three weeks of implantation [21]. While glutaraldehyde is an effective crosslinker, aldehydes unreleased from the reaction can cause toxicity [108,109]. Conversely, N-(3-dimethylaminopropyl)-N′-ethylcarbodiimide hydrochloride (EDC) covalently binds collagen molecules but is not incorporated into the collagen structure [108] and can easily be rinsed out, resulting in improvements in scaffold stability while maintaining cytocompatibility. Crosslinking with EDC alone or in conjunction with N-hydroxysuccinimide (NHS) was utilized in 28% of the studies assessed. This crosslinking strategy has been shown to maintain electrospun collagen stability in vitro for over two weeks and support tissue healing and regeneration in long-term tendon repair studies [71]. Other zero-length crosslinkers have been utilized, including genipin, a naturally occurring crosslinker with low cytotoxicity [68].

A direct comparison of crosslinking electrospun collagen using glutaraldehyde immersion (25% for 24 h), EDC-NHS (20 mM EDC and 10 mM NHS in 90% ethanol for 24 h) and genipin (30 mM genipin in 90% ethanol for 24 h at 37 °C) showed significant increases in hydrated scaffold ultimate tensile strength and elongation with EDC-NHS crosslinking; however, all scaffolds were capable of preventing rapid degradation of the scaffold and supporting MC3T3-E1 cell adhesion and proliferation [19]. In contrast, a direct comparison of electrospun collagen scaffolds crosslinked with EDC-NHS (30 wt % EDC and NHS as 1:1 (*w/w*) in a 9:1 (*v/v*) acetone/water mixture at room temperature for one day) or genipin (0.5 wt % GP at 25 °C for 48 h) showed a marked decrease in MG-63 cell attachment and proliferation on genipin crosslinked scaffolds versus EDC/NHS crosslinked scaffolds [20]. The concentration of genipin utilized in the MG-63 study (~22 mM) was similar to the MC3T3-E1 cell study (30 mM); reduced attachment was observed in the prior study. Extensive rinsing of the scaffolds was performed in the MC3T3-E1 study with 5 rinses in ethanol immediately after crosslinking followed by additional rinses in ethanol (30 min), phosphate-buffered saline (5 exchanges, 15 min. each), sterile PBS (3 exchanges) and medium [19], whereas the MG-63 study reported only “several” rinses in deionized water [20]. These results highlight the need for not only a careful selection of crosslinker chemistry and concentration but also postprocessing/rinsing of the scaffolds to obtain the desired properties with low to no cytotoxicity.

To avoid issues associated with partial solubilization of the scaffolds while immersed in the crosslinking solution, vapor-based and physical crosslinking methods have been utilized. Glutaraldehyde vapor is one of the most widely utilized crosslinking methods (Table 2) and has been shown to enhance the biostability of collagen and collagen blends for in vitro culture up to four weeks [46]. Nevertheless, concerns regarding unreleased aldehydes remain a concern. Physical crosslinking methods, such as ultraviolet irradiation (UV) [20,54,71], Argon laser irradiation [61,62,63] or dehydrothermal treatment (DHT) [26,36,39,76,77,93] are effective ways to improve scaffold stability and mechanics without exposure to potentially cytotoxic agents. UV crosslinking forms bonds at aromatic amino acid residues by initiating the formation of free radicals. UV treatment of electrospun collagen was found to impart stability for in vitro cell culture of up to 28 days with the UV crosslinked scaffolds promoting survival of human bone marrow-derived mesenchymal stem cells and improving cardiac differentiation compared to 2D controls [54]. In a separate study, UV treatment was shown to modestly improve crosslinking and thermal stability versus no treatment [20]. While it did not reach the level of crosslinking achieved by chemical crosslinkers (genipin, transglutaminase and EDC + N-hydroxysuccinimide), it appeared to have better-preserved fiber morphology and porosity after treatment [20]. Dehydrothermal treatment of collagen causes amide and ester bond formation and, when applied to electrospun collagen for 24 h, it has shown excellent preservation of fiber diameter after hydration, increased resistance to degradation in an aqueous environment and under collagenase load compared to no crosslinking, and deeper cell penetration into the scaffold compared to control and EDC crosslinking [36].

Additionally, physical crosslinkers can be used in combination with chemical crosslinkers. Though this can further increase scaffold preparation time and cost, they can be tuned to have an additive effect, resulting in significant scaffold improvements [36]. While separate use of DHT and EDC cross-linkers significantly decreased electrospun collagen scaffold degradation in an aqueous environment and improved mechanics, the combinatorial treatment of the two further improved resistance to degradation and ultimate tensile strength and generally improved cellular metabolic activity in vitro over both no crosslinking and DHT alone [36].

#### 3.3.2. Composition

**Figure 4:**
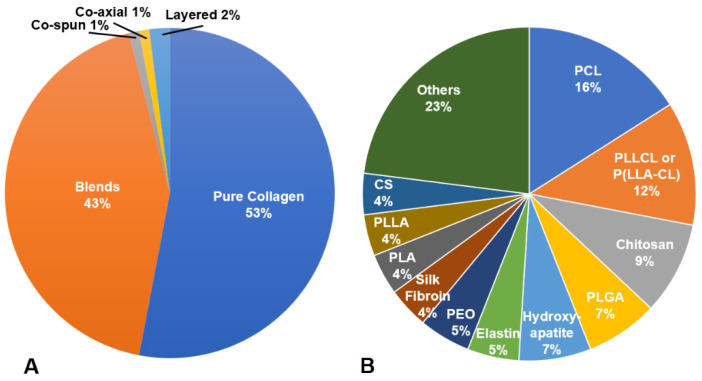
(**A**) Type of collagen-based electrospun scaffolds; (**B**) distribution of materials combined with collagen in electrospun scaffolds.

The use of crosslinkers on electrospun collagen can achieve suitable scaffold stability and resistance to degradation for in vitro culture and in vivo implantation; however, further enhancements to mechanical properties, reductions in degradation rate or optimization of biological response may be desired. Therefore, many techniques have explored to combine collagen with other natural or synthetic polymers or additives via blending, co-electrospinning, electrospinning alternating layers of the components or coating the electrospun fibers [16,41,60,75,80,81]. Within the manuscripts reviewed, only ~43% of the scaffolds investigated pure collagen scaffolds. Blends were most commonly utilized (~57% of total scaffolds), with co-spinning, coaxial electrospinning, layering and coating utilized to a far lesser extent (Figure 4A). Of the compositions studied, poly(L-lactic acid)-co-poly(ε-caprolactone) (PLCL), poly(caprolactone) (PCL), chitosan and polylactic acid (PLA) were the most common polymers/biopolymers utilized in conjunction with collagen, though there were many unique compositions examined once (Figure 4B). In many studies, the combined use of collagen and other polymers was intended to enhance cell attachment, proliferation and or maintain phenotype compared to the synthetic polymer alone. For example, blending of collagen with PLCL significantly enhanced human coronary artery endothelial cell attachment compared to P(LLA-CL) alone [44]. When a blend of PLLA and collagen was compared with co-spinning each solution separately (PLLA in chloroform, collagen in HFP), the blend had higher average ultimate tensile strength than the co-spun materials. Higher ALP activity was also observed from mesenchymal stem cells cultured on the blend versus the co-spun or PLLA controls [41].

**Figure 5:**
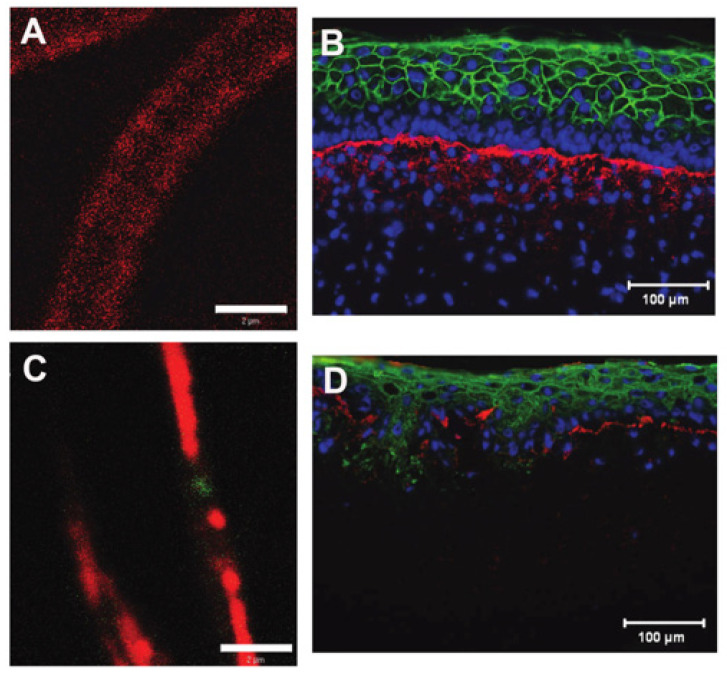
Confocal images of pure collagen (red) (**A**) and collagen (red)-polycaprolactone (PCL) (green) blend electrospun fibers (**C**) showing the segregation of the collagen and PCL components at a total PCL concentration of 30% total polymer mass. Scale bar = 7 µm. These large gelatin and PCL domains within the fiber reduced the ability of the collagen-PCL blend to support the formation of a well-stratified engineered skin (**D**) (blue = DAPI, red= collagen IV, green = involucrin) despite improvements to acellular scaffold strength and resistance to degradation. Pure collagen scaffolds support the development of bilayered engineered skin with a stratified epidermis (**B**). Modified from [75] with permission.

With each blend of chemistry and composition, there is a balance of biological response and physical properties. For example, the blending of collagen type I with chitosan facilitated endothelial and smooth muscle cell attachment but significantly reduced ultimate tensile strength [30]. Blending collagen with polycaprolactone (PCL) prior to electrospinning brought scaffolds closer to the mechanical properties of native tissues, including skin [75]. While this combination was suitable for implantation as a vascular graft [87], the presence of synthetic polymers can have deleterious effects, as in the case of engineered skin where scaffolds with PCL composition of greater than 3% resulted in significantly decreased cell metabolic activity and greater than 10% PCL were unable to support proper development of an epidermis (Figure 5) [75]. In the case of coaxial electrospinning, cells immediately contact the biocompatible collagen shell, though as the scaffold degrades, cells will be exposed to the core material and its degradation products, which can result in late-term cytotoxicity and/or inflammation. Material composition is critical to both scaffold and cell functioning and should be closely evaluated for each specific tissue engineering application.

### 3.4. Tissue Engineering Applications

Electrospun (ES) collagen-based scaffolds have been used in a wide array of tissue engineering applications, both in vitro and in vivo, including facilitating healing after injury, and replacement of skin [26,53,77,93], blood vessels [27,46], bone [65,78], and muscle [17,98], as well as neural [49,60,61,72] tissues. For each of these applications, the composition, crosslinking and architecture of the scaffold is tailored to meet the demands of the target tissue.

#### 3.4.1. Skin Tissue Engineering and Wound Healing

Electrospinning of type I collagen produces scaffolds that is inherently similar to the native ECM within the dermis in chemistry and in microscale architecture; thus, they are often utilized in wound regeneration and skin tissue engineering applications. In situ crosslinked collagen-chitosan nanofibers have been utilized to enhance angiogenesis and epithelialization in a rat scald model [32]. Quaternary ammonium organosilane crosslinked nanofibrous collagen scaffolds facilitated the growth of dermal fibroblasts and inhibited the growth of staphylococcus epidermidis and MRSA [33]. Electrospun collagen-based scaffolds have also been seeded with human dermal fibroblasts prior to grafting to serve as a surrogate dermis. Human dermal fibroblast-seeded ES collagen scaffolds were shown to support the attachment of human cultured epithelial autografts in a full-thickness wound model in athymic mice [110]. Collagen-PLLCL scaffolds have been utilized as a matrix to facilitate mesenchymal stem cell (MSC) differentiation into epithelial cells for epidermal regeneration [53]. Additionally, both human fibroblasts and keratinocytes can be seeded onto electrospun collagen for the development of tissue-engineered skin [39,93]. Electrospun collagen scaffolds supported the development of a stratified epidermis with good barrier function and greater resistance to contraction versus engineered skin fabricated using collagen sponges following grafting to full-thickness wounds in an athymic mouse model [77]. Micropatterning the electrospun collagen-based dermis with a fractional CO_2_ laser mimics the dermal papillae of native tissue and facilitates the formation of rete ridges after the addition of keratinocytes [26] or cultured epithelial autografts [110] for up to four weeks post-grafting. Overall, collagen-based electrospun scaffolds have been observed to serve as a surrogate ECM and enhance re-epithelialization and wound closure.

#### 3.4.2. Cardiovascular Applications

Electrospun collagen is also being explored for cardiovascular tissue applications as it can easily be collected onto a mandrel to form tubes with a wide range of inner and outer diameters for blood vessel engineering or maintained as a sheet for myocardium repair. An electrospun collagen/PCL blend was used as a vascular graft in a rat model, where scaffolds were pre-implanted in the peritoneal cavity for autologous cells to attach and then grafted into the abdominal aorta [84]. Grafts displayed burst pressures greater than 2000 mmHg prior to grafting and maintained mechanical integrity over four weeks of implantation [84]. A collagen/PCL blend seeded with endothelial cells on the lumen and smooth muscle cells on the outside of the tube was successfully grafted in a rabbit aortoiliac bypass model for one month [87]. Electrospun collagen is also being investigated for cardiac patch applications. Human bone marrow-derived stem cell spheroids underwent cardiomyogenesis on electrospun collagen scaffolds for cardiac patch development [54]. Coaxial electrospun collagen-poly(glycerol sebacate) supported survival primary rabbit cardiomyocytes and differentiation of mesenchymal stem cells in a co-culture cardiac patch system [80]. Kitsara et al. found that electrospun collagen supported cardiomyoblast growth in vitro and, when grafted to the ventricle in a dilated cardiomyopathy mouse model, the acellular scaffold elicited no apparent inflammatory response [56]. In the studies assessed, electrospinning collagen-synthetic polymer blends and coaxial scaffolds enhanced scaffold and/or tissue strength while maintaining sufficient biocompatibility to support cell attachment and differentiation.

#### 3.4.3. Neural Applications

As electrospinning can generate scaffolds with high levels of alignment, including radially aligned and unidirectionally aligned, these matrices have been widely utilized for neural regeneration [60,61,62,64,69,72,88]. Tubular, radially aligned collagen/PCL scaffolds were engineered with an SDF1α gradient to promote neural stem cell migration toward the center of the construct to aid in guided nerve regeneration [60]. Unidirectionally aligned pure collagen scaffolds guided neurite outgrowth from dorsal root ganglia and aligned astrocytes when cultured in vitro. These scaffolds were rolled into a tubular conduit and implanted into a hemisection spinal cord defect in mice where neural fiber sprouting was observed after ten days, and maintenance of the aligned tubular structure was observed after 30 days implantation [61]. Electrospun collagen-PLGA tubular nerve conduits (NCs) were utilized to treat a 13 mm defect in the proximal sciatic nerve of rats. The aligned NCs organized the Schwann cells along with the long axis of the fibers and significantly enhanced functional recovery versus randomly aligned NCs as measured using walking gait analysis [72].

#### 3.4.4. Musculoskeletal Applications

For bone engineering applications, many different collagen-based electrospun materials have been studied. Collagen-PCL scaffolds dip-coated in 45S5 bioglass induced osteoblast differentiation and hydroxyapatite crystal formation in vitro [24]. Collagen has been electrospun with other polymers and proteins such as PCL, hydroxyapatite and chitosan to improve biomechanics and tissue function [58,73,90]. A collagen, PCL and hydroxyapatite blend was electrospun and implanted in cortical defects created in rat tibiae [73]. After seven days, new bone was found throughout the scaffold and defect [73]. An electrospun blend of collagen and chitosan applied to full-thickness cranial defects in a rat model supported new growth of bone connecting the scaffold to the defect border, was infiltrated by inflammatory cells, mesenchymal cells and new capillaries, and facilitated bone regeneration over eight weeks [42].

Engineered muscle tissue, where myoblasts were seeded in the lumen of an electrospun collagen tube, was implanted in rat quadriceps and showed fully differentiated myotubes at eight weeks that mimicked native muscle [17]. Aligned ES PCL/collagen was used for diaphragmatic muscle reconstruction, where scaffolds were implanted into a diaphragm defect in rats and supported tissue development and mechanics of grafts similar to native tissue after implantation from two to six months post-grafting [98]. As a potential meniscus repair, electrospun collagen was seeded with cells isolated from human menisci and implanted ex vivo into defects of explanted bovine menisci, resulting in well-integrated neotissue within the defect site [21]. Aligned electrospun collagen was shown to improve fibrillogenesis and biomechanics over no treatment in large Achilles tendon defects in rabbits [71]. Electrospun collagen-poly(L-lactic acid-co-ε-caprolactone) seeded with chondrocytes and implanted subcutaneously in nude mice displayed cartilage-like tissue with Young’s modulus close to that of native cartilage and no cytotoxicity at 12 weeks post-implantation [16].

## 4. Discussion

As collagens are the dominant protein in the extracellular matrix of many tissue and organs, they are a common choice for tissue engineering scaffolds to mimic ECM chemistry. To mimic the structure of the native ECM, electrospinning of collagen is utilized to form nanometric fibers in random or oriented matrices. Though electrospun collagen and collagen-based scaffolds are abundant in the literature, controversy exists regarding how closely this material mimics native collagen and the fabrication/postprocessing methods required to impart stability without toxicity. Prior studies would suggest a complete loss of collagen ultrastructure, including d-banding, following electrospinning [18,111]. However, most studies within this systematic review did not examine collagen ultrastructure and, of the ones that did (eleven total), six showed evidence of D-banding, with the majority of these studies using calfskin as the raw material with HFIP as a solvent using relatively short solubilization times. These studies suggest that the source material, specifically the manner in, which the collagen is isolated, is critical to the collagen ultrastructure and demonstrate that electrospun collagen can be formed with structural fidelity to native collagen though it is unclear what level of biomimicry is needed for successful tissue engineering. A large difference in cell-scaffold interactions, tissue formation and inflammation was observed when electrospun gelatin was compared to electrospun collagen, highlighting the importance of a collagen base material [17]. However, the need to mimic the ultrastructure is not known. Many engineered tissues have been successfully constructed using electrospun collagen (both with and without demonstration of D-banding) and have promoted healing and regeneration in vivo. As these scaffolds are remodeled in vivo, it may not be necessary to fully mimic the structure during the initial phases of tissue fabrication as the ECM will be remodeled one grafted/implanted.

As the degradation rate and mechanical properties of scaffold for tissue engineering play a role in their function and suitability for engraftment/implantation, crosslinking and/or the addition of synthetic polymers has been utilized to enhance enzymatic and mechanical stability. Contradicting reports regarding the toxicity of chemical crosslinkers, including glutaraldehyde, EDC and genipin. Even when very similar concentrations of crosslinker are utilized, outcomes can vary from no observable toxicity to substantial reductions in attachment and proliferation. A notable feature in the crosslinking protocols is the preparation of the scaffold for cell seeding following the initial crosslinking. Studies with extensive rinsing protocols report less apparent toxicity than studies with only a brief mention of rinsing post crosslinking. In contrast, scaffold strength and resistance to degradation were more uniformly improved with the addition of synthetic polymers, whether in the form of blends, coaxial electrospinning or co-electrospinning. Unfortunately, in the blend format, cell attachment and proliferation were often reduced with increasing concentrations of the synthetic polymer. As a result, the use of collagen alone is recommended when the tissue can be protected during in vivo remodeling. In cases where enhanced strength or slow degradation rates are required, the smallest ratio of synthetic polymer to collagen to meet these needs is suggested.

## 5. Conclusions

Electrospun collagen is a versatile matrix for tissue engineering that can be tuned to meet the needs of the target tissue. Ultrastructure can be maintained under specific collagen isolation and solubilization conditions. Irrespective of the presence of D-banding, electrospun collagen has been shown to have distinct advantages over electrospun gelatin and has been utilized to engineer many tissues for use in vitro and in vivo.

## Figures and Tables

**Table 1:** Assessment of D-banding in scaffolds electrospun collagen scaffolds.

Origin	Source	Solvent	Ultrastructure	Solution Injection Rate	Ref.
Calfskin	Extracted in-house	HFP	D-banding observed via TEM	3–7 mL/h	[17]
Calfskin	Sigma-Aldrich	HFP	D-banding observed via TEM	5 mL/h	[22]
Calfskin	Sigma-Aldrich	HFP	D-banding observed via AFM	0.5–1.5 mL/h	[25]
Calfskin	Sigma-Aldrich	HFP	D-banding observed via TEM	2–8 mL/h	[27]
Fish-derived collagen type I	Medira Ltd.	Acetic Acid: DMSO (93:7)	D-banding observed via TEM	0.6 mL/h	[38]
Calfskin	Extracted in-house	HFP	D-banding observed via TEM	Not reported	[86]
Porcine dermis	Extracted in-house	HFP	Maintenance of structure via circular dichroism	0.2 mL/h	[64]
Source not listed	Sichuan Ming-rang Bio-Tech Co. Ltd.	HFP	No D-banding observed via XRD	0.8 mL/h	[30]
Bovine dermis (soluble)	Kensey Nash Corporation	HFP	No D-banding observed via TEM	4 mL/h	[36]
Tilapia skin	Extracted in-house	HFP	No maintenance of structure observed via circular dichroism	1.0 mL/h	[59]

**Table 2:** Crosslinking methods for collagen and collagen-based electrospun scaffolds.

Crosslinker	Delivery	Solvent	Exposure/Concentration	Treatment Time	Ref.
Argon laser irradiation	Irradiation	-	514 nm, 226 mW, spot size of d = 2 cm at RT	100 s	[61,62,63]
BDDGE	Immersion	Ethanol	5% *w*/*v* at RT	7 days at 37 °C	[40]
CaCl_2_ + Ammonium Carbonate	CaCl_2_ in situ, (NH_4_)_2_CO_3_ environmental	-	20 mM Ca^2+^, 5 g (NH_4_)_2_CO_3_ in desiccator at RT	24 h	[34]
Citric Acid (+/− glycerol)	In situ	-	5 wt % of collagen wt +/− 3% glycerol at RT	-	[56,57]
DHT	Environmental	-	Vacuum at 140 °C	24 h	[26,36,39,76,77,93]
EDC	Immersion	90–100% Ethanol	5–200 mM, 5 *w*/*v*% at RT or 37 °C	4 h–7 days	[26,32,36,39,40,47,49,75,76,77,93]
EDC+NHS	Immersion	90–100% Ethanol, 90% Acetone	30–600 mM EDC, 10–600 mM NHS at 4 °C—RT	4 h–24 h	[19,20,35,49,50,51,52,60,71,106]
Genipin	Immersion	90–100% Ethanol, 90% Acetone, 95–100% Isopropanol	3.5–30 mM, 0.5–10 *w*/*w*% at RT—37 °C	24 h–5 days	[19,20,49,68,74,88]
Glutaraldehyde	Vapor	-	0.5–50 *v*% at RT	15 min–3 days	[17,19,22,27,29,30,31,37,41,43,46,48,51,65,66,67,68,69,72,78,80,81,82,86,97,99,100,101,103]
Glutaraldehyde	Immersion	1X PBS, Distilled Water, Ethanol	0.25–40 *v*% at RT	1–19 h	[21,23,49,58,70]
HMDI,1,6-diisocyanatohexane	Immersion	Isopropanol	10 *v*%	2 h	[85]
Phosphoric Acid	Vapor	-	-	24 h	[95]
Quaternary ammonium organosilane (QOS) + Ammonium carbonate	QOS in situ, (NH_4_)_2_CO_3_ environmental	-	0.1–10 *w*% Silane, 5 g (NH_4_)_2_CO_3_ in desiccator at RT	48 h for (NH_4_)_2_CO_3_	[33]
Thermal treatment	Environmental	-	150°C	1.5–2.5 h	[51,54]
Transglutaminase	Immersion	Phosphate buffer	5000:1 *w*/*w* TG:Collagen at RT	Overnight	[20]
UV	Irradiation	-	365 nm UVA (3 mW/cm^2^,d = 50mm, with 0.1% riboflavin), 254 nm, 253.7 nm (30 W)	30 min–1 h	[20,54,71]

## Supplementary Materials

The following are available online at https://www.mdpi.com/2306-5354/8/3/39/s1, Table S1: Summary of collagen source and composition of scaffolds utilized in the assessed studies. Major outcomes were noted along with tissue engineering application. If no specific tissue engineering application was listed in the respective manuscript, “tissue engineering” is listed.

## References

[1] Horbert V., Xin L., Foehr P., Brinkmann O., Bungartz M., Burgkart R.H., Graeve T., Kinne R.W. (2018). In vitro analysis of cartilage regeneration using a collagen type I hydrogel (CaReS) in the bovine cartilage punch model. Cartilage.

[2] Hosseini Y., Verbridge S.S., Agah M. (2014). Bio-inspired microstructures in collagen type I hydrogel. J. Biomed. Mater. Res. Part A.

[3] Boyce S.T. (2004). Fabrication, quality assurance, and assessment of cultured skin substitutes for treatment of skin wounds. Biochem. Eng. J..

[4] Iejima D., Saito T., Uemura T. (2003). A collagen-phosphophoryn sponge as a scaffold for bone tissue engineering. J. Biomater. Sci. Polym. Ed..

[5] Sumita Y., Honda M.J., Ohara T., Tsuchiya S., Sagara H., Kagami H., Ueda M. (2006). Performance of collagen sponge as a 3-D scaffold for tooth-tissue engineering. Biomaterials.

[6] Sill T.J., Von Recum H.A. (2008). Electrospinning: Applications in drug delivery and tissue engineering. Biomaterials.

[7] Maurmann N., Sperling L.-E., Pranke P. (2018). Electrospun and Electrosprayed Scaffolds for Tissue Engineering. Adv. Exp. Med. Biol..

[8] Sell S.A., McClure M.J., Garg K., Wolfe P.S., Bowlin G.L. (2009). Electrospinning of collagen/biopolymers for regenerative medicine and cardiovascular tissue engineering. Adv. Drug Deliv. Rev..

[9] Gao X., Han S., Zhang R., Liu G., Wu J. (2019). Progress in electrospun composite nanofibers: Composition, performance and applications for tissue engineering. J. Mater. Chem. B.

[10] Kishan A.P., Cosgriff-Hernandez E.M. (2017). Recent advancements in electrospinning design for tissue engineering applications: A review. J. Biomed. Mater. Res. Part A.

[11] Ameer J.M., Pr A.K., Kasoju N. (2019). Strategies to Tune electrospun scaffold porosity for effective cell response in tissue engineering. J. Funct. Biomater..

[12] Walters B., Stegemann J. (2014). Strategies for directing the structure and function of three-dimensional collagen biomaterials across length scales. Acta Biomater..

[13] Fullana M.J., Wnek G.E. (2012). Electrospun collagen and its applications in regenerative medicine. Drug Deliv. Transl. Res..

[14] Sofi H.S., Ashraf R., Beigh M.A., Sheikh F.A., Crusio W.E., Dong H., Lambris J.D., Radeke H.H., Rezei N. (2018). Scaffolds fabricated from natural polymers/composites by electrospinning for bone tissue regeneration. Advances in Experimental Medicine and Biology.

[15] Holmes J., Molnar J., Shupp J., Hickerson W., King B.T., Foster K., Cairns B., Carter J. (2019). Demonstration of the safety and effectiveness of the RECELL^®^ System combined with split-thickness meshed autografts for the reduction of donor skin to treat mixed-depth burn injuries. Burns.

[16] He X., Fu W., Feng B., Wang H., Liu Z., Yin M., Wang W., Zheng J. (2013). Electrospun collagen–poly (L-lactic acid-co-ε-caprolactone) membranes for cartilage tissue engineering. Regen. Med..

[17] Jha B.S., Ayres C.E., Bowman J.R., Telemeco T.A., Sell S.A., Bowlin G.L., Simpson D.G. (2011). Electrospun Collagen: A Tissue engineering scaffold with unique functional properties in a wide variety of applications. J. Nanomater..

[18] Zeugolis D.I., Khew S.T., Yew E.S., Ekaputra A.K., Tong Y.W., Yung L.-Y.L., Hutmacher D.W., Sheppard C., Raghunath M. (2008). Electro-spinning of pure collagen nano-fibres—Just an expensive way to make gelatin?. Biomaterials.

[19] Luo X., Guo Z., He P., Chen T., Li L., Ding S., Li H. (2018). Study on structure, mechanical property and cell cytocompatibility of electrospun collagen nanofibers crosslinked by common agents. Int. J. Biol. Macromol..

[20] Torres-Giner S., Gimeno-Alcañiz J.V., Ocio M.J., Lagaron J.M. (2008). Comparative Performance of electrospun collagen nanofibers cross-linked by means of different methods. ACS Appl. Mater. Interfaces.

[21] Baek J., Sovani S., Glembotski N.E., Du J., Jin S., Grogan S.P., D’Lima D.D. (2016). Repair of Avascular meniscus tears with electrospun collagen scaffolds seeded with human cells. Tissue Eng. Part A.

[22] Matthews J.A., Wnek G.E., Simpson D.G., Bowlin G.L. (2002). Electrospinning of Collagen nanofibers. Biomacromolecules.

[23] Baek J., Sovani S., Choi W., Jin S., Grogan S.P., D’Lima D.D. (2018). Meniscal tissue engineering using aligned collagen fibrous scaffolds: Comparison of different human cell sources. Tissue Eng. Part A.

[24] Balasubramanian P., Roether J.A., Schubert D.W., Beier J.P., Boccaccini A.R. (2015). Bi-layered porous constructs of PCL-coated 45S5 bioactive glass and electrospun collagen-PCL fibers. J. Porous Mater..

[25] Barrientos I.J.H., Paladino E., Szabó P., Brozio S., Hall P.J., Oseghale C.I., Passarelli M.K., Moug S.J., Black R.A., Wilson C.G. (2017). Electrospun collagen-based nanofibres: A sustainable material for improved antibiotic utilisation in tissue engineering applications. Int. J. Pharm..

[26] Blackstone B.N., Malara M.M., Baumann M.E., McFarland K.L., Supp D.M., Powell H.M. (2020). Fractional CO_2_ laser micropatterning of cell-seeded electrospun collagen scaffolds enables rete ridge formation in 3D engineered skin. Acta Biomater..

[27] Eugene D.B. (2004). Electrospinning collagen and elastin: Preliminary vascular tissue engineering. Front. Biosci..

[28] Carlisle C., Coulais C., Guthold M. (2010). The mechanical stress–strain properties of single electrospun collagen type I nanofibers. Acta Biomater..

[29] Casper C.L., Yang W., Farach-Carson M.C., Rabolt J.F. (2007). Coating electrospun collagen and gelatin fibers with perlecan domain i for increased growth factor binding. Biomacromolecules.

[30] Chen Z., Wang P., Wei B., Mo X., Cui F. (2010). Electrospun collagen–chitosan nanofiber: A biomimetic extracellular matrix for endothelial cell and smooth muscle cell. Acta Biomater..

[31] Chong C., Wang Y., Fathi A., Parungao R., Maitz P.K., Li Z. (2019). Skin wound repair: Results of a pre-clinical study to evaluate electropsun collagen–elastin–PCL scaffolds as dermal substitutes. Burns.

[32] Deng A., Yang Y., Du S., Yang S. (2018). Electrospinning of in situ crosslinked recombinant human collagen peptide/chitosan nanofibers for wound healing. Biomater. Sci..

[33] Dhand C., Balakrishnan Y., Ong S.T., Dwivedi N., Venugopal J.R., Harini S., Leung C.M., Low K.Z.W., Loh X.J., Beuerman R.W. (2018). Antimicrobial quaternary ammonium organosilane cross-linked nanofibrous collagen scaffolds for tissue engineering. Int. J. Nanomed..

[34] Dhand C., Ong S.T., Dwivedi N., Diaz S.M., Venugopal J.R., Navaneethan B., Fazil M.H., Liu S., Seitz V., Wintermantel E. (2016). Bio-inspired in situ crosslinking and mineralization of electrospun collagen scaffolds for bone tissue engineering. Biomaterials.

[35] Dong B., Arnoult O., Smith M.E., Wnek G.E. (2009). Electrospinning of collagen nanofiber scaffolds from benign solvents. Macromol. Rapid Commun..

[36] Drexler J.W., Powell H.M. (2011). Dehydrothermal crosslinking of electrospun collagen. Tissue Eng. Part C Methods.

[37] Drobota M., Gradinaru L.M., Vlad S., Bargan A., Butnaru M., Angheloiu M., Aflori M. (2020). Preparation and characterization of electrospun collagen based composites for biomedical applications. Materials.

[38] Elamparithi A., Punnoose A.M., Kuruvilla S. (2015). Electrospun type 1 collagen matrices preserving native ultrastructure using benign binary solvent for cardiac tissue engineering. Artif. Cells Nanomed. Biotechnol..

[39] Ebersole G., Anderson P., Powell H. (2010). Epidermal differentiation governs engineered skin biomechanics. J. Biomech..

[40] Fiorani A., Gualandi C., Panseri S., Montesi M., Marcacci M., Focarete M.L., Bigi A. (2014). Comparative performance of collagen nanofibers electrospun from different solvents and stabilized by different crosslinkers. J. Mater. Sci. Mater. Med..

[41] Gonçalves F., Bentini R., Burrows M.C., Carreira A.C.O., Kossugue P.M., Sogayar M.C., Catalani L.H. (2015). Hybrid membranes of plla/collagen for bone tissue engineering: A Comparative Study of scaffold production techniques for optimal mechanical properties and osteoinduction ability. Materials.

[42] Guo S., He L., Yang R., Chen B., Xie X., Jiang B., Weidong T., Ding Y. (2019). Enhanced effects of electrospun collagen-chitosan nanofiber membranes on guided bone regeneration. J. Biomater. Sci. Polym. Ed..

[43] Hartman O., Zhang C., Adams E.L., Farach-Carson M.C., Petrelli N.J., Chase B.D., Rabolt J.F. (2009). Microfabricated electrospun collagen membranes for 3-D cancer models and drug screening applications. Biomacromolecules.

[44] He W., Yong T., Teo W.E., Ma Z., Ramakrishna S. (2005). Fabrication and endothelialization of collagen-blended biodegradable polymer nanofibers: Potential vascular graft for blood vessel tissue engineering. Tissue Eng..

[45] He X., Fu W., Feng B., Wang H., Liu Z., Yin M., Wang W., Zheng J. (2013). Electrospun Collagen/Poly (L-lactic acid-co-ε-caprolactone) Hybrid nanofibrous membranes combining with sandwich construction model for cartilage tissue engineering. J. Nanosci. Nanotechnol..

[46] Heydarkhan-Hagvall S., Schenke-Layland K., Yang J.Q., Heydarkhan S., Xu Y., Zuk P.A., MacLellan W.R., Beygui R.E. (2008). Human adipose stem cells: A potential cell source for cardiovascular tissue engineering. Cells Tissues Organs.

[47] Hsu Y.-M., Chen C.-N., Chiu J.-J., Chang S.-H., Wang Y.-J. (2009). The effects of fiber size on MG63 cells cultured with collagen based matrices. J. Biomed. Mater. Res. Part B Appl. Biomater..

[48] Huang C., Chen R., Ke Q., Morsi Y., Zhang K., Mo X. (2011). Electrospun collagen–chitosan–TPU nanofibrous scaffolds for tissue engineered tubular grafts. Colloids Surfaces B Biointerfaces.

[49] Huang G.P., Shanmugasundaram S., Masih P., Pandya D., Amara S., Collins G., Arinzeh T.L. (2014). An investigation of common crosslinking agents on the stability of electrospun collagen scaffolds. J. Biomed. Mater. Res. Part A.

[50] Jia W., Li M., Kang L., Gu G., Guo Z., Chen Z. (2019). Fabrication and comprehensive characterization of biomimetic extracellular matrix electrospun scaffold for vascular tissue engineering applications. J. Mater. Sci..

[51] Jiang Q., Reddy N., Zhang S., Roscioli N., Yang Y. (2012). Water-stable electrospun collagen fibers from a non-toxic solvent and crosslinking system. J. Biomed. Mater. Res. Part A.

[52] Jie Y., Cai Z., Li S., Xie Z., Ma M., Huang X. (2017). Hydroxyapatite nucleation and growth on collagen electrospun fibers controlled with different mineralization conditions and phosvitin. Macromol. Res..

[53] Jin G., Prabhakaran M.P., Ramakrishna S. (2011). Stem cell differentiation to epidermal lineages on electrospun nanofibrous substrates for skin tissue engineering. Acta Biomater..

[54] Joshi J., Brennan D., Beachley V., Kothapalli C.R. (2018). Cardiomyogenic differentiation of human bone marrow-derived mesenchymal stem cell spheroids within electrospun collagen nanofiber mats. J. Biomed. Mater. Res. Part A.

[55] Kempf M., Miyamura Y., Liu P.-Y., Chen A.C.-H., Nakamura H., Shimizu H., Tabata Y., Kimble R.M., McMillan J.R. (2011). A denatured collagen microfiber scaffold seeded with human fibroblasts and keratinocytes for skin grafting. Biomaterials.

[56] Kitsara M., Joanne P., Boitard S.E., Ben Dhiab I., Poinard B., Menasché P., Gagnieu C., Forest P., Agbulut O., Chen Y. (2015). Fabrication of cardiac patch by using electrospun collagen fibers. Microelectron. Eng..

[57] Kung F.H., Sillitti D., Shrirao A.B., Shreiber D.I., Firestein B.L. (2018). Collagen nanofibre anisotropy induces myotube differentiation and acetylcholine receptor clustering. J. Tissue Eng. Regen. Med..

[58] Lee H., Yeo M., Ahn S., Kang D.-O., Jang C.H., Lee H., Park G.-M., Kim G.H. (2011). Designed hybrid scaffolds consisting of polycaprolactone microstrands and electrospun collagen-nanofibers for bone tissue regeneration. J. Biomed. Mater. Res. Part B Appl. Biomater..

[59] Li D., Gao Y., Wang Y., Yang X., He C., Zhu M., Zhang S., Mo X. (2019). Evaluation of biocompatibility and immunogenicity of micro/nanofiber materials based on tilapia skin collagen. J. Biomater. Appl..

[60] Li X., Li M., Sun J., Zhuang Y., Shi J., Guan D., Chen Y., Dai J. (2016). Radially aligned electrospun fibers with continuous gradient of SDF1α for the guidance of neural stem cells. Small.

[61] Liu T., Houle J.D., Xu J., Chan B.P., Chew S.Y. (2012). Nanofibrous collagen nerve conduits for spinal cord repair. Tissue Eng. Part A.

[62] Liu T., Teng W.K., Chan B.P., Chew S.Y. (2010). Photochemical crosslinked electrospun collagen nanofibers: Synthesis, characterization and neural stem cell interactions. J. Biomed. Mater. Res. Part A.

[63] Liu T., Xu J., Chan B.P., Chew S.Y. (2011). Sustained release of neurotrophin-3 and chondroitinase ABC from electrospun collagen nanofiber scaffold for spinal cord injury repair. J. Biomed. Mater. Res. Part A.

[64] Liu X., Dan W., Ju H., Dan N., Gong J. (2015). Preparation and evaluation of a novel pADM-derived micro- and nano electrospun collagen membrane. RSC Adv..

[65] Lotfi G., Shokrgozar M.A., Mofid R., Abbas F.M., Ghanavati F., Baghban A.A., Yavari S.K., Pajoumshariati S. (2015). Biological evaluation (in vitro and in vivo) of bilayered collagenous coated (nano electrospun and solid wall) chitosan membrane for periodontal guided bone regeneration. Ann. Biomed. Eng..

[66] Lu H., Chen W.-J., Xing Y., Ying D.-J., Jiang B. (2009). Design and preparation of an electrospun biomaterial surgical patch. J. Bioact. Compat. Polym..

[67] Matthews J.A., Boland E.D., Wnek G.E., Simpson D.G., Bowlin G.L. (2003). Electrospinning of collagen type II: A feasibility study. J. Bioact. Compat. Polym..

[68] Mekhail M., Wong K.K.H., Padavan D.T., Wu Y., O’Gorman D.B., Wan W. (2011). Genipin-Cross-linked Electrospun collagen fibers. J. Biomater. Sci. Polym. Ed..

[69] Mohamadi F., Ebrahimi-Barough S., Nourani M.R., Derakhshan M.A., Goodarzi V., Nazockdast M.S., Farokhi M., Tajerian R., Majidi R.F., Ai J. (2017). Electrospun nerve guide scaffold of poly(ε-caprolactone)/collagen/nanobioglass: An in vitro study in peripheral nerve tissue engineering. J. Biomed. Mater. Res. Part A.

[70] Newton D., Mahajan R., Ayres C., Bowman J.R., Bowlin G.L., Simpson D.G. (2009). Regulation of material properties in electrospun scaffolds: Role of cross-linking and fiber tertiary structure. Acta Biomater..

[71] Oryan A., Moshiri A., Meimandi A.P., Silver I.A. (2013). A long-term in vivo investigation on the effects of xenogenous based, electrospun, collagen implants on the healing of experimentally-induced large tendon defects. J. Musculoskelet. Neuronal Interact..

[72] Ouyang Y., Huang C., Zhu Y., Fan C., Ke Q. (2013). Fabrication of seamless electrospun collagen/PLGA conduits whose walls comprise highly longitudinal aligned nanofibers for nerve regeneration. J. Biomed. Nanotechnol..

[73] Phipps M.C., Clem W.C., Grunda J.M., Clines G.A., Bellis S.L. (2012). Increasing the pore sizes of bone-mimetic electrospun scaffolds comprised of polycaprolactone, collagen I and hydroxyapatite to enhance cell infiltration. Biomaterials.

[74] Polk S., Sori N., Thayer N., Kemper N., Maghdouri-White Y., Bulysheva A.A., Francis M.P. (2018). Pneumatospinning of collagen microfibers from benign solvents. Biofabrication.

[75] Powell H.M., Boyce S.T. (2009). Engineered human skin fabricated using electrospun Collagen–PCL blends: Morphogenesis and mechanical properties. Tissue Eng. Part A.

[76] Powell H.M., McFarland K.L., Butler D.L., Supp D.M., Boyce S.T. (2010). Uniaxial strain regulates morphogenesis, gene expression, and tissue strength in engineered skin. Tissue Eng. Part A.

[77] Powell H.M., Supp D.M., Boyce S.T. (2008). Influence of electrospun collagen on wound contraction of engineered skin substitutes. Biomaterials.

[78] Qiao X., Russell S.J., Yang X., Tronci G., Wood D.J. (2015). Compositional and in vitro evaluation of nonwoven type i collagen/poly-dl-lactic acid scaffolds for bone regeneration. J. Funct. Biomater..

[79] Râpă M., Gaidău C., Stefan L.M., Matei E., Niculescu M., Berechet M.D., Stanca M., Tablet C., Tudorache M., Gavrilă R. (2020). New Nanofibers Based on Protein By-Products with Bioactive Potential for Tissue Engineering. Materials.

[80] Ravichandran R. (2013). Cardiogenic differentiation of mesenchymal stem cells on elastomeric poly (glycerol sebacate)/collagen core/shell fibers. World J. Cardiol..

[81] Sharifi-Aghdam M., Faridi-Majidi R., Derakhshan M.A., Chegeni A., Azami M. (2017). Preparation of collagen/polyurethane/knitted silk as a composite scaffold for tendon tissue engineering. Proc. Inst. Mech. Eng. Part H J. Eng. Med..

[82] Shields K.J., Beckman M.J., Bowlin G.L., Wayne J.S. (2004). Mechanical properties and cellular proliferation of electrospun collagen type II. Tissue Eng..

[83] Shoae-Hassani A., Mortazavi-Tabatabaei S.A., Sharif S., Seifalian A.M., Azimi A., Samadikuchaksaraei A., Verdi J. (2013). Differentiation of human endometrial stem cells into urothelial cells on a three-dimensional nanofibrous silk-collagen scaffold: An autologous cell resource for reconstruction of the urinary bladder wall. J. Tissue Eng. Regen. Med..

[84] Shojaee M., Wood K.B., Moore L.K., Bashur C.A. (2017). Peritoneal pre-conditioning reduces macrophage marker expression in collagen-containing engineered vascular grafts. Acta Biomater..

[85] Slater S.C., Beachley V., Hayes T., Zhang D., Welsh G.I., Saleem M.A., Mathieson P.W., Wen X., Su B., Satchell S.C. (2011). An in vitro model of the glomerular capillary wall using electrospun collagen nanofibres in a bioartificial composite basement membrane. PLoS ONE.

[86] Telemeco T., Ayres C., Bowlin G., Wnek G., Boland E., Cohen N., Baumgarten C., Mathews J., Simpson D. (2005). Regulation of cellular infiltration into tissue engineering scaffolds composed of submicron diameter fibrils produced by electrospinning. Acta Biomater..

[87] Tillman B.W., Yazdani S.K., Lee S.J., Geary R.L., Atala A., Yoo J.J. (2009). The in vivo stability of electrospun polycaprolactone-collagen scaffolds in vascular reconstruction. Biomaterials.

[88] Timnak A., Gharebaghi F.Y., Shariati R.P., Bahrami S.H., Javadian S., Emami S.H., Shokrgozar M.A. (2011). Fabrication of nano-structured electrospun collagen scaffold intended for nerve tissue engineering. J. Mater. Sci. Mater. Electron..

[89] Türker E., Yildiz Ü.H., Yildiz A.A. (2019). Biomimetic hybrid scaffold consisting of co-electrospun collagen and PLLCL for 3D cell culture. Int. J. Biol. Macromol..

[90] Venugopal J., Low S., Choon A.T., Kumar T.S.S., Ramakrishna S. (2008). Mineralization of osteoblasts with electrospun collagen/hydroxyapatite nanofibers. J. Mater. Sci. Mater. Med..

[91] Wang S., Banerjee A., Matarlo B., Arinzeh T.L., Ophir Z., Jaffe M., Collins G. (2012). Structure and morphology of electrospun collagen blends. Bioinspired Biomim. Nanobiomater..

[92] Wei K., Li Y., Mugishima H., Teramoto A., Abe K. (2011). Fabrication of core-sheath structured fibers for model drug release and tissue engineering by emulsion electrospinning. Biotechnol. J..

[93] Willard J.J., Drexler J.W., Das A., Roy S., Shilo S., Shoseyov O., Powell H.M. (2013). Plant-derived human collagen scaffolds for skin tissue engineering. Tissue Eng. Part A.

[94] Wu T., Zheng H., Chen J., Wang Y., Sun B., Morsi Y., El-Hamshary H., Al-Deyab S.S., Chen C., Mo X. (2016). Application of a bilayer tubular scaffold based on electrospun poly (l-lactide-co-caprolactone)/collagen fibers and yarns for tracheal tissue engineering. J. Mater. Chem. B.

[95] Wu Z., Kong B., Liu R., Sun W., Mi S. (2018). Engineering of corneal tissue through an aligned PVA/collagen composite nanofibrous electrospun scaffold. Nanomaterials.

[96] Yao Q., Zhang W., Chunyi S., Chen J., Shao C., Fan X., Fu Y. (2017). Electrospun collagen/poly(L-lactic acid‑co‑ε‑caprolactone) scaffolds for conjunctival tissue engineering. Exp. Ther. Med..

[97] Yu P., Guo J., Li J., Shi X., Wang L., Chen W., Mo X. (2017). Repair of skin defects with electrospun collagen/chitosan and fibroin/chitosan compound nanofiber scaffolds compared with gauze dressing. J. Biomater. Tissue Eng..

[98] Zhao W., Ju Y.M., Christ G., Atala A., Yoo J.J., Lee S.J. (2013). Diaphragmatic muscle reconstruction with an aligned electrospun poly(ε-caprolactone)/collagen hybrid scaffold. Biomaterials.

[99] Zhao X., Gao J., Hu X., Guo H., Wang F., Qiao Y., Wang L. (2018). Collagen/Polyethylene oxide nanofibrous membranes with improved hemostasis and cytocompatibility for wound dressing. Appl. Sci..

[100] Zhong S., Teo W.E., Zhu X., Beuerman R., Ramakrishna S., Yung L.Y.L. (2005). Formation of collagen−glycosaminoglycan blended nanofibrous scaffolds and their biological properties. Biomacromolecules.

[101] Li Z., Zhou Y., Yao H., Wang J., Wang D., Liu Q. (2015). Greener synthesis of electrospun collagen/hydroxyapatite composite fibers with an excellent microstructure for bone tissue engineering. Int. J. Nanomed..

[102] Zhu B., Li W., Chi N., Lewis R.V., Osamor J., Wang R. (2017). Optimization of glutaraldehyde vapor treatment for electrospun collagen/silk tissue engineering scaffolds. ACS Omega.

[103] Silvipriya K.S., Kumar K.K., Bhat A.R., Kumar B.D., John A., Lakshmanan P. (2015). Collagen: Animal sources and biomedical application. J. Appl. Pharm. Sci..

[104] Stein H., Wilensky M., Tsafrir Y., Rosenthal M., Amir R., Avraham T., Ofir K., Dgany O., Yayon A., Shoseyov O. (2009). production of bioactive, post-translationally modified, heterotrimeric, human recombinant type-i collagen in transgenic tobacco. Biomacromolecules.

[105] Gautieri A., Vesentini S., Redaelli A., Buehler M.J. (2011). Hierarchical structure and nanomechanics of collagen microfibrils from the atomistic scale up. Nano Lett..

[106] Gutsmann T., Fantner G.E., Kindt J.H., Venturoni M., Danielsen S., Hansma P.K. (2004). Force spectroscopy of collagen fibers to investigate their mechanical properties and structural organization. Biophys. J..

[107] Bürck J., Heissler S., Geckle U., Ardakani M.F., Schneider R., Ulrich A.S., Kazanci M. (2013). Resemblance of electrospun collagen nanofibers to their native structure. Langmuir.

[108] Duan X., Sheardown H. (2005). Crosslinking of collagen with dendrimers. J. Biomed. Mater. Res. Part A.

[109] Khor E. (1997). Methods for the treatment of collagenous tissues for bioprostheses. Biomaterials.

[110] Malara M.M., Blackstone M.B.N., Baumann M.M.E., Bailey J.K., Supp D.M., Powell H.M. (2020). Cultured epithelial autograft combined with micropatterned dermal template forms rete ridges in vivo. Tissue Eng. Part A.

[111] Sizeland K.H., Hofman K.A., Hallett I.C., Martin D.E., Potgieter J., Kirby N.M., Hawley A., Mudie S.T., Ryan T.M., Haverkamp R.G. (2018). Nanostructure of electrospun collagen: Do electrospun collagen fibers form native structures?. Materials.

