# Supplementary material for: Collagen-Based Electrospun Materials for Tissue Engineering: A Systematic Review
Source: Bioengineering (Basel). 2021 Mar 18;8(3):39. doi: 10.3390/bioengineering8030039 (PMC8003061; doi:10.3390/bioengineering8030039)
Supplement: Supplementary file 1 [file bioengineering-08-00039-s001.pdf]

Supplemental Table 1. Summary of collagen source and composition of scaffolds utilized in the assessed studies. Major outcomes were noted along with tissue engineering application. If no specific tissue engineering application was listed in the respective manuscript, "tissue engineering" is listed.

Authors/Ref	Electrospun Scaffold Composition	Outcomes	Application
Baek J *et al*[23]	Bovine collagen type I	• Addition of electrospun collagen scaffolds to layered hydrogel supported meniscus-like neotissue formation<br>• Increased mechanical strength observed compared to avascular and acellular scaffolds	Meniscus regeneration
Baek J *et al*[21]	Bovine collagen type I	• Electrospun collagen scaffolds seeded with human meniscus avascular cells improved neotissue formation and integration in an explanted bovine meniscus avascular zone defect model versus untreated defects or acelluar scaffolds	Meniscus regeneration
Balasubramanian P *et al*[24]	Calf type I collagen blended with PCL	• Immersion of collagen:PCL blend scaffolds in SBF for 21 days resulted in hydroxyapatite formation on both layers	Interface TE applications (bone/cartilage)
Barrientos IJH *et al*[25]	Calf type I collagen blended with PLA	• PLA-collagen-LEVO samples resulted in sustained drug release of LEVO *in vitro* as opposed to a burst release in PLA-LEVO	Drug delivery
Blackstone BN *et al*[26]	Bovine collagen type I	• Electrospun collagen scaffolds seeded with human primary fibroblasts were laser pattered to form wells in surface<br>• Addition of primary human keratinocytes to the engineered dermis results in skin with rete ridge-like structures<br>• Grafting of ridged engineered skin to full-thickness wounds improved epidermal barrier formation, epidermal proliferation and basement membrane production compared to flat controls.	Skin, wound healing
Boland ED *et al*[27]	Calf type I collagen blended with elastin	• Collagen-elastin blends supported smooth muscle cell, endothelial cell and fibroblast adhesion and viability.<br>• A three layered vessel was observed with distinct zones of intima, media, and adventitia	Vascular
Carlisle CR *et al*[28]	Calf type I collagen	• Small strain bending modulus was 2.82±0.4 GPa; extensibility 33±3%; maximum stress 25±3MPa<br>• Modulus softened as strain increased<br>• Permanent deformation and significant energy loss above 2% strain.	Tissue engineering
Casper CL *et al*[29]	Calf type I collagen or gelatin coated with perlecan	• Collagen was found to bind both Perlecan domain I and the heparin-BSA-biotin complex more readily than gelatin	Tissue engineering
Chen ZG *et al*[30]	Collagen type I alone or blended with chitosan	• Addition of chitosan reduced ultimate tensile strength<br>• Higher concentration of chitosan reduced endothelial cell proliferation at early time points, no difference by day 7	Tissue engineering (further studies for blood

Supplemental Table 1. Summary of collagen source and composition of scaffolds utilized in the assessed studies. Major outcomes were noted along with tissue engineering application. If no specific tissue engineering application was listed in the respective manuscript, "tissue engineering" is listed.

		• Collagen-chitosan (4:1, 1:1) and pure chitosan scaffolds were found to be optimal for cell proliferation and with ideal levels of surface area and porosity	vessel and nerve)
Chong C *et al*[31]	Rat tail collagen type I blended with elastin, PCL or both elastin and PCL	• Highest strength observed with the collagen-PCL scaffold<br>• Tripolymer scaffold supported HaCaT and primary human fibroblast growth for 28 days<br>• Collagen-elastin-PCL scaffolds showed higher cell attachment on day 1, as well as greater cell proliferation versus collagen-elastin-PCL on days 7 and 21<br>• In murine wounds, electrospun collagen–elastin–PCL scaffolds promoted keratinocyte and fibroblast proliferation, tissue integration and accelerated early-stage angiogenesis as compared to Integra	Skin
Deng A *et al*[32]	Recombinant human collagen type I blended with PEO and chitosan	• *In situ*-crosslinked scaffolds resulted in more uniform distribution of NIH 3T3 cells compared to immersion-crosslinked scaffolds<br>• In SD rat scald model, *in situ* and immersion crosslinked scaffolds sped wound healing rates compared to non-treated controls<br>• Small blood vessel formation and formation of hair follicles were observed in the *in situ* crosslinked scaffold group	Wound healing
Dhand C *et al*[33]	Bovine collagen type I with and without QOS added	• Peak stress and Young's Modulus was highest in the collagen-only scaffolds<br>• QOS crosslinking was toxic at high concentrations<br>• Collagen+0.1%QOS scaffolds resulted in the highest total cell number at day 9 of culture.	Wound healing
Dhand C *et al*[34]	Bovine type I collagen with and without catecholamines and $CaCl_2$	• Addition of catecholamines and $CaCl_2$ resulted in no toxicity<br>• Collagen+catecholamine+Ca^{2+} scaffolds after $(NH_4)_2CO_3$ exposure exhibited relatively high fetal osteoblast spreading as well as cell penetration	Bone
Dong B *et al*[35]	Bovine type I collagen	• FTIR spectra suggest that the triple helical structure of collagen was conserved after dissolution and electrospinning	Tissue engineering
Drexler JW *et al*[36]	Bovine type I collagen	• Physical and chemical crosslinking reduced degradation, improved mechanical properties and supported fibroblast growth and penetration into electrospun collagen scaffolds<br>• EDC and DHT+EDC groups exhibited higher fibroblast metabolism than DHT alone; however DHT treatment resulted in greater fibroblast migration into the scaffold	Tissue engineering (dermal regeneration)
Drobota M *et al*[37]	Calf type I collagen alone and with PET blended	• Blend improved mechanical properties of scaffold and supported cell viability for 72hr	Tissue engineering

Supplemental Table 1. Summary of collagen source and composition of scaffolds utilized in the assessed studies. Major outcomes were noted along with tissue engineering application. If no specific tissue engineering application was listed in the respective manuscript, "tissue engineering" is listed.

		• MCF-7 density appeared greater on collagen-PET blend than collagen alone	(dermal regeneration)
Ebersole GC *et al*[39]	Bovine type I collagen	• UTS and stiffness in engineered skin increases with culture time and correlated with epidermal differentiation<br>• No difference in mechanical properties of engineered dermis observed with time in culture	Skin
Elamparithi *et al*[38]	Fish-derived collagen type I	• Electrospun fibers exhibit D-banding<br>• L6 rat skeletal myoblasts cultured on collagen scaffolds expressed muscle-specific markers including desmin and actin<br>• More rapid beating of primary neonatal rat ventricular cardiomyocytes was observed on nanofiber scaffold versus 2D culture at culture days 9-17<br>• Cardiomyocytes were observed throughout the scaffold	Cardiovascular
Fiorani A *et al*[40]	Bovine type I collagen	• Collagen scaffolds crosslinked with EDC contained a very low amount of triple helix but still supported MSC attachment<br>• BDDGE maintained fiber morphology in electrospun collagen	Tissue engineering
Gonçalves F *et al*[41]	Collagen-PLLA blend (1:1)<br>Collagen and PLLA co-spun	• Blend scaffold had the largest tensile strength compared to co-spun scaffolds<br>• Collagen-PCL blends exhibited optimal mechanical properties and the highest capacity for osteogenic differentiation in human MSCs	Bone
Guo *et al*[42]	Bovine tendon collagen type I with and without chitosan	• Electrospun collagen maintained individual fiber morphology with less fiber bonding and a slower degradation rate than collagen-chitosan blends<br>• Collagen-chitosan blends promoted more rapid human periodontal ligament cell proliferation<br>• In a rat skull defect model, greater bone alkaline phosphatase and osteocalcin was detected with the collagen-chitosan scaffolds after 4 weeks of implantation, no difference between scaffold groups was observed after 8 weeks	Bone
Hartman O *et al*[43]	Calf type I collagen	• Collagen scaffolds spun using HFP resulted in larger fiber diameters than TFE and subsequently enhanced migration into the scaffolds<br>• 3D culture environment of electrospun collagen promoted spheroid-like clusters of C4-2B cells compared to 2D cultures	In vitro cancer model and drug screening
He W *et al*[44]	Calf type I collagen blended with P(LLA-CL)	• Collagen-blends reduced strength and modulus compared to P(LLA-CL) alone<br>• Collagen-blended polymer nanofibers enhanced the viability, spreading, and attachment of human coronary artery ECs compared to P(LLA-CL) scaffolds	Tissue engineering (blood vessel)

Supplemental Table 1. Summary of collagen source and composition of scaffolds utilized in the assessed studies. Major outcomes were noted along with tissue engineering application. If no specific tissue engineering application was listed in the respective manuscript, “tissue engineering” is listed.

He X *et al*[45]	Collagen type I blended with PLCL	• Primary rabbit chondrocytes cultured on collagen-PLCL scaffolds produced ECM after 3 days of culture<br>• In a subcutaneous implantation model, chondrocyte-seeded scaffolds exhibited cartilage-like tissue growth as indicated by type II collagen and GAG deposition<br>• Young's modulus of constructs reached to 83% of native rabbit auricular cartilage at 12 weeks post-implantation<br>• GAG content increased over time and reached native levels	Cartilage
He X *et al*[16]	Collagen type I blended with PLCL	• Modulus of chondrocyte-seeded collagen-PLCL scaffolds reached 32, 55, and 77% of native cartilage modulus at at weeks 4, 8, and 12-post implantation, respectively<br>• Histological analysis confirmed the collagen-PLCL construct facilitated growth of cartilage-like tissue in vivo	Cartilage
Heydarkhan-Hagvall S *et al*[46]	Calf type I collagen with elastin	• Collagen-elastin scaffold provided a matrix for human adipose stem cells growth and differentiation into endothelial cells	Cardiovascular
Hsu YM *et al*[47]	Bovine type I collagen	• Collagen nanofibers promoted MG-63 cell interaction with matrices by providing suitably rough nanometric surface<br>• Viability of MG-63 cells grown on ES nanofibers was ~70% higher than cells grown on polystyrene or a monomeric collagen-coated surface<br>• MG-63 cells on smaller fibers (50-200, 200-500 nm) migrated more quickly than those on larger fibers (500-1000 nm)	Tissue engineering
Huang C *et al*[48]	Collagen type I blended with chitosan and TPU	• Randomly, parallel and perpendicularly aligned scaffolds electrospun<br>• Porcine iliac artery endothelial cells and Schwann cells attached and proliferated on all collagen–chitosan–TPU blended nanofibrous scaffolds<br>• Schwann cells proliferated ~25% higher on randomly aligned scaffolds	Blood vessel and nerve repair
Huang GP *et al*[49]	Bovine tendon type I	• Multiple crosslinkers were assessed<br>• Genipin (5%) resulted in the highest modulus and ultimate tensile strength versus genipin (1%), EDC, EDC+NHS and glutaraldehyde<br>• Cytotoxicity when human MSCs were cultured on glutaraldehyde crosslinked collagen scaffolds<br>• MSCs were well-spread on EDC-NHS scaffolds, with increased cell numbers at day 7	Tissue engineering
Jha BS *et al*[17]	Calf-skin corium type I collagen or gelatin	• Electrospun collagen scaffolds and electrospun gelatin scaffolds were used for multiple tissue engineering applications	Tissue engineering (blood vessels,

Supplemental Table 1. Summary of collagen source and composition of scaffolds utilized in the assessed studies. Major outcomes were noted along with tissue engineering application. If no specific tissue engineering application was listed in the respective manuscript, "tissue engineering" is listed.

		• ECs, osteoblasts, dermal fibroblasts were all successfully cultured on electrospun collagen<br>• Osteoblasts exhibited greater proliferation and confluency on electrospun gelatin compared to electrospun collagen; however, the electrospun collagen facilitated the formation of phase bright, hydroxyapatite crystals<br>• No fibrosis was apparent on engineered muscle implants made of collagen, whereas gelatin based implants became necrotic	bone, wound healing and muscle)
Jia W *et al*[50]	Collagen type I with hyaluronic acid oligosaccharides covalently bonded	• Collagen–hyaluronic acid scaffolds promoted porcine iliac artery endothelial cell proliferation<br>• Collagen-hyaluronic acid fibers had significantly higher numbers of endothelial cells at culture day 7 than collagen alone	Vascular
Jiang Q *et al*[51]	Bovine type I collagen	• Multiple crosslinking strategies were compared<br>• Citric acid crosslinking appeared better than glutaraldehyde for NIH 3T3 cell adhesion and growth<br>• Native collagen conformation was retained after electrospinning with benign solvent	Tissue engineering
Jie Y *et al*[52]	Rat tail type I collagen	• Electrospun collagen in simulated body fluid results in hydroxyapatite deposition on the surface<br>• With static mineralization, flaky spheres of minerals were observed while they were hexagonal with dynamic mineralization	Bone
Jin G *et al*[53]	Collagen type I blended with PLLCL or PLLCL alone	• Collagen-PLLCL blended nanofibers exhibited a higher tensile modulus than that of pure PLLCL<br>• Ultimate tensile stress for PLLCL and collagen-PLLCL were similar<br>• Proliferation of human bone-derived mesenchymal stem cells was higher on collagen:PLLCL blended scaffolds than PLLCL<br>• Differentiation into epithelial cells was observed on the collagen:PLLCL scaffolds after 15 days in culture	Skin
Joshi J *et al*[54]	Calf type I collagen	• Scaffolds supported culture and cardiomyogenesis of bone marrow mesenchymal stem cell spheroids<br>• Spheroids cultured on scaffolds exhibited migration after 24 hours and spreading across the scaffold by days 12 and 28<br>• Cells exhibited alignment of 0-20 degrees after 28 days in culture, characteristic of cardiomyocytes	Myocardium
Kempf M *et al*[55]	Bovine type I collagen	• Scaffold facilitated growth and proliferation of HaCaTs, fibroblasts and normal human epithelial keratinocytes (NHEK)<br>• Stratified and differentiated epidermal-like layers were observed in fibroblast + NHEK seeded scaffolds	Skin

Supplemental Table 1. Summary of collagen source and composition of scaffolds utilized in the assessed studies. Major outcomes were noted along with tissue engineering application. If no specific tissue engineering application was listed in the respective manuscript, "tissue engineering" is listed.

		• In an immunodeficient (SCID) excisional wound model, dermal thickening was lower in wounds treated with skin equivalents compared to untreated wounds	
Kitsara M *et al*[56]	Atelocollagen, acid fibrous collagen, basic fibrous collagen	• Thermal treatment of scaffolds enhanced scaffold stiffness and resistance to degradation<br>• H9c2 cardiomyoblasts adhered to and proliferated on collagen scaffolds<br>• Following a left lateral thoracotomy in a rat, acellular collagen scaffolds were sutured on the left ventricle<br>• Scaffolds were biocompatible with their degradation rate a function of the crosslinker concentration (higher levels led to slower degradation)	Cardiac patch
Kung FH *et al*[57]	Bovine type I collagen	• Fabricated aligned and random electrospun collagen scaffolds<br>• Observed fewer nuclei in myotubes on aligned collagen fibers than on random<br>• More multinucleated myotubes were observed on aligned scaffolds with greater acetylcholine receptor clustering	Musculo-skeletal
Lee H *et al*[58]	Collagen type I collagen layered within 3D plotted PCL scaffold	• Increased modulus and tensile strength in layered scaffolds versus PCL alone<br>• MG-63 cells were not observed in PCL-only constructs<br>• MG-63 cell proliferation was lower with large pore sizes compared to small pore sizes in the collagen-PCL layered scaffolds	Bone
Li D *et al*[59]	Tilapia skin type I collagen blended with PLGA	• L929 attached to and were viable on collagen-PLGA scaffolds<br>• Good biocompatibility and low immunogenicity were observed when implanted subcutaneously in BALB/c mice	Tissue engineering
Li X *et al*[60]	Collagen blended with PCL	• Radially aligned collagen-PCL fibers exhibited higher tensile strength compared to randomly aligned fibers<br>• Radially aligned tubular conduit that promotes cell migration from periphery to center<br>• An increased gradient of SDF1a and radial alignment of fibers enhanced cell migration towards the center of the scaffolds, , indicating potential for guiding nerve regeneration	Neural
Liu T *et al*[61]	Rat tail type I collagen	• Fiber morphology (random or aligned) determined cell elongation<br>• Neurite growth from DRG oriented along aligned fibers, however in random fibers, neurites extended radially<br>• Elongated morphology of astrocytes observed in aligned scaffolds<br>• Proliferation of astrocytes significantly lower on collagen scaffolds as compared to 2D controls	Neural/Spinal cord repair

Supplemental Table 1. Summary of collagen source and composition of scaffolds utilized in the assessed studies. Major outcomes were noted along with tissue engineering application. If no specific tissue engineering application was listed in the respective manuscript, "tissue engineering" is listed.

		• In an acute, rat hemi-section spinal cord injury model, tubular collagen scaffolds were still visible 10 days post-implantation<br>• Cell penetration was observed in structures regardless of fiber orientation with decreased cell presence observed at day 30, indicating reduced acute inflammatory response	
Liu T *et al*[62]	Rat tail type I collagen	• Acetic acid-electrospun, photochemical crosslinked collagen nanofibers<br>• Morphology of murine neural stem cells (C17.2) were rounded on collagen nanofibers at day 3, but formed a confluent layer by day 7.	Neural
Liu T *et al*[63]	Rat tail type I collagen	• Biofunctional nanofiber constructs that may provide topographical signals and multiple biochemical cues that can promote nerve regeneration	Neural/Spinal cord repair
Liu X *et al*[64]	Porcine collagen type I	• Tensile strength was higher for porcine-derived electrospun collagen (PDEC) scaffolds compared to collagen-derived electrospun scaffolds<br>• PDEC scaffolds possessed higher content of α-helix structure but less β-turn structure	Tissue engineering
Lotfi G *et al*[65]	Calf skin type I collagen alone and spun on top of a chitosan membrane	• Collagen nanofiber-chitosan membrane has significantly more new bone deposition than the chitosan or collage-chitosan membranes in a rabbit skull defect model<br>• Higher ALPase activity and calcium content was observed in the collagen-nanofiber-chitosan membrane group<br>• Collagen nanofiber-chitosan membrane defects showed bone formation in all directions, as compared to localized bone formation in defect borders in other groups	Bone
Lu H *et al*[66]	Rat tail type I collagen	• Electrospun collagen solutions between 8-20% collagen in HFP<br>• Modulus of scaffold in wet conditions similar to porcine cardiac pericardium<br>• Collagen scaffolds facilitated HUVEC adhesion and infiltration	Cardiovascular
Luo X *et al*[19]	Rat tail type I collagen	• Multiple crosslinking strategies investigated<br>• Highest ultimate tensile strength, modulus and elongation at failure was observed with EDC-NHS crosslinking<br>• MC3T3-E1 cell proliferation was lowest on scaffolds crosslinked with glutaraldehyde vapor, while the highest amount of proliferation observed when EDC-NHS was utilized	Tissue engineering
Matthews JA *et al*[67]	Chicken sternal cartilage type II collagen	• Collagen type II scaffolds were electrospun<br>• Chondrocytes were observed to penetrate the scaffold matrix, and scaffold remodeling was observed	Tissue engineering (cartilage)

Supplemental Table 1. Summary of collagen source and composition of scaffolds utilized in the assessed studies. Major outcomes were noted along with tissue engineering application. If no specific tissue engineering application was listed in the respective manuscript, "tissue engineering" is listed.

Matthews JA *et al*[22]	Calf skin type I collagen, human placental type I and type III collagen	• Electrospun collagen scaffolds ES collagen scaffolds facilitated attachment and proliferation of aortic smooth muscle cells<br>• Aortic smooth muscle cells densely populated the matrix with migration observed deep into the matrix	Tissue engineering
Mekhail M *et al*[68]	Rat tail type I collagen	• Fibroblasts attached to uncrosslinked and genipin crosslinked scaffolds<br>• Cells on uncrosslinked scaffolds showed a flat morphology, whereas genipin-crosslinked scaffolds resulted in stellate cell morphologies	Tissue engineering
Mohamadi F *et al*[69]	Rat tail type I collagen, PCL and nanobioglass blend	• Electrospun collagen-PCL-nanobioglass (NBG) scaffolds promoted primary human endometrial stem cell adhesion (hEnSCs) and proliferation<br>• hEnSC activity was significantly higher on the electrospun scaffold on day 7 versus 2D controls	Neural
Newton D *et al*[70]	Calf skin corium type I collagen	• Ultimate strength increased as crosslinking % increased<br>• Increased % crosslinking with glutaraldehyde resulted in robust scaffolds, with strain inducing changes in fiber alignment	Tissue engineering
Oryan A *et al*[71]	Bovine tendon type I collagen	• Acellular tendon-like bioimplant (electrospun collagen scaffold) was biocompatible, biodegradable and appeared suitable for clinical use (rabbit tendon defect)	Tendon
Ouyang Y *et al*[72]	Collagen type I blended with PLGA	• Alignment increased strength compared to random fibers<br>• Schwann cells grown on aligned collagen/PCL scaffolds exhibited parallel alignment to fibers, and were observed to have a bipolar morphology, typical of Schwann cells<br>• In rat sciatic nerve defect model, uniaxially aligned fibers resulted in a significantly higher nerve conduction velocity and distal compound motor action potential at week 12 compared to randomly oriented fibers	Neural
Phipps *et al*[73]	Calf skin type I collagen with PCL and hydroxyapatite with and without co-spun PEO fibers	• Addition of sacrificial PEO fibers enhanced porosity of scaffolds<br>• MSC penetration into scaffolds with PEO was significant improved<br>• In murine calvaria explant model, the use of sacrificial PEO fibers greatly improved endogenous cell infiltration into the scaffold	Bone
Polk S *et al*[74]	Calf corium atelocollagen type I alone or blended with PDLLA	• Pneumatospun collagen exhibited greater strength compared to electrospun collagen<br>• No significant difference was observed for modulus of elasticity or strain at break<br>• No difference in human adipose-derived stem cell viability was observed between scaffolds	Tissue engineering with a focus on ligament, tendon, and nerve repair

Supplemental Table 1. Summary of collagen source and composition of scaffolds utilized in the assessed studies. Major outcomes were noted along with tissue engineering application. If no specific tissue engineering application was listed in the respective manuscript, "tissue engineering" is listed.

Powell HM *et al*[75]	Bovine type I collagen alone or blended with PCL	• Scaffold strength and stiffness were increased with increasing PCL content<br>• Primary human fibroblast and keratinocyte metabolism and proliferation were lower in scaffolds with >30% PCL content.<br>• Blends with higher concentrations of PCL (>30% of total polymer content) resulted in poor skin stratification	Skin
Powell HM *et al*[76]	Bovine type I collagen	• Engineered skin was formed using electrospun collagen scaffolds<br>• Engineered skin could be cultured under strain<br>• Ultimate tensile strength and linear stiffness increased in skin cultured under static strain with the exception of 40% applied strain<br>• Upregulation of genes for ECM and adhesion molecules occurred in 20% strain group after 3 days in culture	Skin
Powell HM *et al*[77]	Bovine type I collagen	• Engineered skin was formed using lyophilized collagen sponges or electrospun collagen<br>• Cell proliferation and tissue anatomy was similar between scaffolds at all time points *in vitro*<br>• Less overall graft contraction was observed in when electrospun collagen scaffolds compared to lyophilized scaffolds	Skin
Qiao X *et al*[78]	Rat tail type I collagen blended with PDLLA	• PDLLA/collagen promoted greater human bone marrow stromal cell proliferation and osteogenic differentiation compared to PDLLA/gelatin scaffolds	Bone
Râpă M *et al*[79]	Bovine type I collagen	• Electrospun keratin hydrolysate (KH), rabbit collagen glue(RCG), and collagen scaffolds were fabricated<br>• Significant cytotoxicity was observed in the KH and RCG groups at higher concentrations	Tissue engineering
Ravichandran R *et al*[80]	Collagen type I alone or coaxial with PGS	• Co-axial electrospinning of PGS and collagen increased strain at failure but decreased the modulus compared to collagen alone<br>• Cardiac cell/MSC proliferation and adhesion was significantly higher on PGS/collagen compared to collagen alone	Cardiac
Sharifi-Aghdam M *et al*[81]	Rat tail collagen alone or blended with PU	• Increasing collagen content from 25 to 75% increased ultimate strength (2.01±1.3 to 0.45±0.1 MPa) and % elongation (113±20 to 15±2%)<br>• Scaffolds with greater collagen content resulted in higher cell viability on day 3<br>• L929 cell viability decreased on all scaffolds after culture day three with greater viability losses observed in the PU only scaffold	Tendon
Shields KJ *et al*[82]	Chicken sternal type II collagen	• Tubular constructs of collagen type II were formed via electrospinning	Cartilage

Supplemental Table 1. Summary of collagen source and composition of scaffolds utilized in the assessed studies. Major outcomes were noted along with tissue engineering application. If no specific tissue engineering application was listed in the respective manuscript, "tissue engineering" is listed.

		• Human articular chondrocytes infiltrated, adhered to, and proliferate on the scaffolds	
Shoae-Hassani A *et al*[83]	Human collagen type V alone or blended with silk fibroin	• Human endometrial stem cells (EnSCs) took on endothelial-like shape when cultured on collagen-silk blends<br>• EnSC adhesion and proliferation higher on collagen-blend compared to other scaffolds<br>• Cell-seeded 3D nanofibrous collagen-silk scaffolds potential use in bladder wall reconstruction in women	Urinary bladder
Shojaee M *et al*[84]	Calf skin type I collagen blended with PCL or PCL alone	• Expression of Acta2 and Eln was greater on 10% collagen blend versus 25%<br>• Higher collagen content (25%) resulted in greater intimal thickening versus 10% and controls in a rat abdominal aorta model	Blood vessels
Slater SC *et al*[85]	Calf type I collagen blended with micro-photo-electroformed nickel mesh	• Electrospun scaffold for in vitro tissue engineering of glomerular capillary for study of both the physiology of normal glomerular filtration and of its disruption in glomerular disease<br>• Culture of glomerular endothelial cells (GEnCs) and podocytes increase cell density versus culturing alone<br>• Resistance of inserts was significantly higher for layers on both sides as opposed to a single layer of GEnC	Kidney
Telemeco TA *et al*[86]	Calf skin corium type I collagen, gelatin, PGA, PLA and PGA-PLA co-polymer (all alone)	• Electrospun collagen tubular constructs promoted cell migration and capillary formation to a greater extent than other materials studies<br>• Collagen scaffolds were fully infiltrated with cells within 7 days of implantation with no indication of fibrotic encapsulation<br>• PGA delaminated from surrounding tissue after 7 days *in vivo*<br>• PGA-based implants exhibited a fibrotic zone with increased cell infiltration	Tissue engineering
Tillman BW *et al*[87]	Calf skin collagen type I blended with PCL	• Collagen-PCL electrospun scaffold supported the adhesion of sheep endothelial progenitor cells to create a confluent monolayer<br>• Smooth muscle cells infiltrated the scaffolds and formed a multilayer configuration on the surface of the scaffolds<br>• In a rabbit aortoiliac bypass model, electrospun grafts remained patent with no significant change in tensile strength or graft diameter and pulsatile flow observed in 7 or the 8 grafts examined	Blood vessel
Timnak A *et al*[88]	Calf skin type I collagen alone or blended with chondroitin sulfate	• Orienting fibers increased ultimate tensile strength (5.2±0.6 MPa) compared to random (0.6±0.1 MPa)<br>• Crosslinking with 2.5 mM genipin for 5 days resulted in highest average cell viability for both fibroblasts and SK-N-MC human neuroblastoma cells<br>• Parallel alignment of cells to fibers was observed.	Neural

Supplemental Table 1. Summary of collagen source and composition of scaffolds utilized in the assessed studies. Major outcomes were noted along with tissue engineering application. If no specific tissue engineering application was listed in the respective manuscript, "tissue engineering" is listed.

		• Nanostructured porous collagen-glycosaminoglycan scaffold is a potential cell carrier in nerve tissue engineering	
Torres-Giner S *et al*[20]	Calf skin type I collagen	• Compared multiple crosslinking methods<br>• MG-63 proliferation increased with time for samples treated with EDC/NHS and transglutaminase versus genipin or UV	Tissue engineering
Türker E *et al*[89]	Calf skin type I collagen blended with PVP or blended with PLLCL, PLLCL alone, PLLCL/PVP blend,	• Ultimate tensile strength was highest for the PLLCL/collagen scaffold<br>• NIH 3T3 cell viability increased with collagen amount in scaffold	Tissue engineering
Venugopal J *et al*[90]	Collagen type I blended with hydroxyapatite	• TE Mineralization was increased to 56% in collagen/HA scaffolds compared to collagen only<br>• Alizarin red S staining indicated mineralization by osteoblasts after 10 days<br>• Calcium and phosphorous levels were higher in collagen/HA scaffolds than collagen alone	Bone
Wang S *et al*[91]	Bovine tendon type I collagen blended with PLLA	• Investigated morphology of blended fibers, where PLLA formed sheath and collagen became core as product of electrospinning parameters/materials	Tissue engineering
Wei K *et al*[92]	Bovine placenta type I collagen blended with PLGA	• Collagen-PLGA fibers exhibited an increased tensile strength compared to PLGA alone<br>• Osteoblast adhesion was significantly higher on collagen/PLGA compared to PLGA alone<br>• Composite scaffold could support the early stages of osteoblast behavior as well as the immediate/late stages	Bone
Willard JJ *et al*[93]	Bovine dermal type I collagen, plant-derived human collagen	• Tensile strength was lower in plant-derived human collagen scaffolds versus bovine-derived collagen scaffolds<br>• Well-stratified engineered skin formed with scaffolds made from both collagen sources<br>• Production of IL-1β was increased in THP-1 cells on bovine collagen scaffolds compared to plant-derived human collagen	Skin
Wu T *et al*[94]	Fish skin type I collagen blended with (P(LLA-CL) or P(LLA-CL) alone	• Tat tracheal epithelial cells and rat tracheal chondrocytes were seeded onto inner and outer layers, respectively, of bilayered tubular scaffolds (BLTS) to form cell-seeded bilayered tubular scaffolds (CS-BLTS)	Trachea

Supplemental Table 1. Summary of collagen source and composition of scaffolds utilized in the assessed studies. Major outcomes were noted along with tissue engineering application. If no specific tissue engineering application was listed in the respective manuscript, "tissue engineering" is listed.

		• Epithelial cell and chondrocyte adhesion and proliferation was significantly enhanced in the collagen-P(LLA-CL) versus P(LLA-CL) alone<br>• CS-BLTS were also embedded in the fascia beside the sternocleidomastoid muscle for 2 weeks to create a prevascularized BLTS (PV-BLTS)<br>• All groups were implanted into a tracheal defect model, capillary regeneration was observed in the PV-BLTS and CS-BLTS at 1 and 2 weeks post-implantation	
Wu Z *et al*[95]	Porcine skin type I collagen alone or blended with PVA	• Mechanical properties of the scaffold improved after addition of PVA<br>• Human keratocytes (HKs) and human corneal epithelial cells (HCECs) adhered to and proliferated on random and aligned collagen-PVA scaffolds<br>• Cells on the random scaffold had lower proliferation on random scaffolds compared to aligned scaffolds	Cornea
Yao Q *et al*[96]	Collagen type I alone or blended with PLCL	• Growth of B4G12 human corneal endothelial cell line and murine conjunctival epithelial cells on collagen/PCL scaffolds maintained a polygonal morphology<br>• Cells were positive for cytokeratin 4 and cytokeratin 9<br>• Inflammatory expression was not significantly different between scaffold and control TCP	Conjunctival epithelium repair
Yu PJ *et al*[97]	Collagen type I blended with chitosan	• On collagen-chitosan scaffolds fibroblasts adhered and exhibited fusiform morphology while keratinocytes cultured on these scaffolds were tightly packed with a cobblestone morphology<br>• Wounds treated with collagen-chitosan scaffold had more rapid wound closure than gauze controls as measured by wound surface area	Skin/wound healing
Zhao W *et al*[98]	Bovine type I collagen blended with PCL	• Scaffolds implanted in the central left hemi-diaphragmatic defect in rats were infiltrated with cells at 1-2 months post implantation with neo-muscle tissue formation at 4 and 6 months<br>• Tensile strength and elasticity of retrieved implants were different than controls at 1 month but equivalent at later time points	Muscle
Zhao X *et al*[99]	Bovine tendon type I collagen blended with PEO	• Glutaraldehyde vapor crosslinked scaffolds promoted blood clotting (70% clotted within 10min)<br>• Scaffolds supported HUVEC adhesion and proliferation	Skin/wound healing
Zhong S *et al*[100]	Calf skin type I with chondroitin sulfate	• Rabbit conjunctiva fibroblasts were more proliferative on crosslinked collagen-chondroitin sulfate scaffolds versus crosslinked collagen scaffolds at day 7	Tissue engineering

Supplemental Table 1. Summary of collagen source and composition of scaffolds utilized in the assessed studies. Major outcomes were noted along with tissue engineering application. If no specific tissue engineering application was listed in the respective manuscript, "tissue engineering" is listed.

Zhou Y *et al*[101]	Calf skin type I with hydroxyapatite	• The addition of hydroxyapatite nanoparticles increased the tensile strength<br>• Human myeloma cells (U2-OS) adhered to and proliferated on collagen-hydroxyapatite scaffolds<br>• ALP activity increased on collagen-hydroxyapatite scaffolds compared to controls.	Bone
Zhu B *et al*[102]	Calf skin type I collagen alone or blended with silk	• Addition of silk increased tensile strength and modulus<br>• Glutaraldehyde (GA) crosslinking tuned mechanical properties and degradation<br>• Undifferentiated human decidua parietalis placental stem cells cultured on 0 and 6 hour GA-crosslinked collagen tightly adhered to and elongated along fibers<br>• Cells on 48 hr GA-crosslinked collagen were rounded and not polarized<br>• On silk fibers, cells were less polarized and exhibited a round or polygonal morphology regardless of GA treatment time	Tissue engineering
